# Stir-Frying of Chinese Cabbage and Pakchoi Retains Health-Promoting Glucosinolates

**DOI:** 10.1007/s11130-017-0646-x

**Published:** 2017-11-13

**Authors:** Probo Y. Nugrahedi, Teresa Oliviero, Jenneke K. Heising, Matthijs Dekker, Ruud Verkerk

**Affiliations:** 1 Food Quality and Design Group, Department of Agrotechnology and Food Sciences, Wageningen University, PO Box 17, 6700 AA Wageningen, The Netherlands; 2 Department of Food Technology, Soegijapranata Catholic University, Jl. Pawiyatan Luhur IV/1 Bendan Duwur, Semarang, 50234 Indonesia

**Keywords:** Glucosinolate, Stir-fry, Chinese cabbage, Pakchoi

## Abstract

Stir-frying is a cooking method, originating from Asia, in which food is fried in small amount of very hot oil. Nowadays in many other parts of the world stir-frying is a very popular method to prepare vegetables, because it is fast and fried vegetables are tasty. However, the retention of phytochemicals like the health-beneficial glucosinolates in *Brassica* vegetables is less explored for stir-frying in comparison to other cooking methods. This study investigates the retention of glucosinolates in Chinese cabbage (*Brassica rapa* ssp. *pekinensis*) and pakchoi (*Brassica rapa* ssp. *chinensis*) as affected by stir-frying at various cooking durations and temperatures. Stir-frying experiments were performed at set pan temperatures ranging from 160 to 250 °C for a duration of 1 to 8 min. Results showed that aliphatic glucobrassicanapin is the most abundant glucosinolate identified in fresh Chinese cabbage and pakchoi, contributing for 48 and 63% of the total glucosinolate content, respectively, followed by glucoiberin and gluconapin. Stir-frying retains the glucosinolates even at the highest temperature applied. Such retention is explained by the quick inactivation of the glucosinolate-hydrolytic enzyme myrosinase during the first minutes of frying, and by the thermal stability of the glucosinolates at those temperature/time conditions. Moreover, due to the absence of a separate water phase, leaching losses did not occur, in contrast to what is observed when boiling *Brassica* vegetables. These results show that stir-frying may be a suitable health-beneficial cooking option that prevents the loss of glucosinolates.

## Introduction

Stir-frying, a popular method to prepare vegetables in Southeast Asian countries [1], is becoming more common nowadays elsewhere in the world. Stir-frying is a quick food preparation method by heat transfer from a hot pan surface to foods, using a small amount of cooking oil. *Brassica* vegetables, including Chinese cabbage (*Brassica rapa* ssp. *pekinensis*) and pakchoi (*Brassica rapa* ssp. *chinensis*), are commonly prepared by this technique. Intake of *Brassica* vegetables was reported to have an inverse association with the risk of developing certain cancers in various epidemiological studies [2]. Glucosinolates (GSs), secondary metabolites present in *Brassica* vegetables, have been widely investigated to play an important role in this health promoting property. Isothiocyanates, one class of GS breakdown products, have been reported to have the ability to prevent many chronic diseases [2]. A GS is a *β*-d-thioglucoside-N-hydroxysulfate with a variable side-chain group, which is derived mainly from methionine, tryptophan, or phenylalanine. The GS can be classified either as aliphatic, aromatic, or indolic [3]. In intact plant tissue GSs are stored in compartments that are physically separated from compartments containing myrosinase enzyme (thioglycosidase EC 3.2.1.147). Upon tissue damage, GSs can come into contact with and be hydrolysed by myrosinase, producing a range of breakdown products including isothiocyanates [4]. Previous studies have indicated that the GS-myrosinase system is modified during thermal processing of *Brassica* vegetables due to inactivation of myrosinase, thermal breakdown of GSs and their hydrolysis products, loss of enzymatic cofactors, cell lysis and subsequent leaching of GSs and their derivatives into the cooking medium [5]. The extent of these losses probably depends on the duration and type of heat treatment, the degree of cell lysis, and the vegetable matrix [6, 7]. Many domestic preparation methods of *Brassica* vegetables have been extensively studied in detail and reviewed [5]. While, only limited information is available on the effect of stir-frying on GS content in *Brassica* vegetables. Also, some conflicting results were reported. On one side, the retention of total GSs and most of individual GS content in green cabbage, broccoli, Brussels sprouts, and cauliflower during stir-frying was observed [8, 9]. On the other side, stir-frying of broccoli and red cabbage was reported to reduce total GSs content by 58 and 77%, respectively [10, 11]. These differences might be due to specific stir-frying conditions applied and the effect of heating on the extraction efficiency of the GSs [5]. Very little information is available on GS changes during stir-frying. Different time and temperature combinations of stir-frying are hypothesized to affect the GS content at various extents. It is also important to notice, that the same GSs present in different *Brassica* vegetables, showed different thermal degradation rate, depending on the type of vegetable [7, 12], suggesting that the different plant-matrix can influence the thermal degradation of GS. Even different genetic lines (doubled haploid populations) of the same *Brassica* variety, were reported to show different GS thermal degradation rates [13]. The present study aims to investigate the stability of GSs in two *Brassica rapa* vegetables, *i.e*., Chinese cabbage and pakchoi, as affected by stir-frying at various heating times and temperatures.

## Materials and Method

### Sample Preparation

A batch of Chinese cabbage and a batch of pakchoi were purchased from a local greengrocer (Wageningen, the Netherlands). For each batch, stems were cut off to have homogenous samples and the leaves were chopped in 1–2 cm strips. Then the chopped leaves were divided into equal portions of 150 g. Each portion consisted of leaves pooled from different vegetables. These followed by either stir-frying or freezing in liquid nitrogen immediately. All samples were then subjected to GS analysis.

### Stir-Frying

Each sample was stir-fried for 1, 2, 4, or 8 min using an electrical frying pan (PZ-2964, Tristar Europe BV) set at 160, 200, 225 or 250 °C. Each preparation was performed in duplicate. Ten mL of sunflower oil (Cap D’or) was poured into the pre-heated pan and heated to the set temperature. Then, 150 g vegetables were added and stirred continuously. The temperature of the pan during stir-frying was regularly monitored with an infra-red thermometer (scan-356, tservice). Upon stir-frying, vegetables were frozen immediately using liquid nitrogen. Then, the samples were freeze-dried (Christ Alpha 1–4 LD plus) and grinded and kept at −20 °C.

### Glucosinolate Analysis

GSs determination was performed in duplicate as described by Verkerk et al. [14]. Briefly, 0.2 g of the freeze dried sample was added to 2.4 mL hot methanol 70% solution (Vol%) and 0.2 mL of 3 mM glucotropaeolin solution as internal standard. Each sample was incubated in a water bath for 20 min at 75 °C and mixed every 5 min. After centrifugation (10 min at 2500 rpm) supernatant was collected and the pellet was re-extracted twice with 2 mL of hot methanol following similar procedure. All supernatants were collected and desulphated on a 1.5 cm DEAE Sephadex A-25 anion exchange column. Sulfatase enzyme (S9626, Sigma) was added to the column and it was incubated overnight at room temperature. The desulpho-GSs were eluted with Millipore water and filtered over a 0.45 μm filter and analysed by HPLC equipped with a LiChrospher® 100 RP-18 column (5 μm, 250 mm × 4.6 mm) (Merck, Darmstadt, Germany) at a flow rate of 1 mL/min (injection volume of 20 μL). Detection was performed at λ = 229 nm. HPLC gradient: (A) water and (B) acetonitrile, from 0 to 2 min, 0% B; from 2 to 7.5 min, 0–8% B; from 7.5 to 14 min, 8–25% B; from 14 to 18 min, 25% B; from 18 to 20 min, 25–0% B; from 20 to 25 min, 0% B as post-run. Each GS was identified by its UV spectra and quantified by using the response factor relative to the internal standard glucotropaeolin. The total GS content was calculated as the sum of all identified GSs.

## Results and Discussion

### Stir-Frying Temperature

The temperature of the pan varied with time and location on the pan surface during stir-frying. Before adding the vegetables the pan surface was at the set temperature. After addition of the vegetables the temperature dropped within one minute with 10 (160 °C) to 70 °C (250 °C), after two minutes the surface temperature had increased again to the mentioned average temperatures for each setting. Therefore, the temperature of the pan decreased quickly upon the addition of the vegetables followed by an increase during stir-frying. Due to this phenomenon the resulting average temperatures during stir-frying varied considerably less (measured pan surface temperatures ranging from 152 to 198 °C) than the temperature prior to stir-frying.

### Glucosinolates in Chinese Cabbage and Pakchoi

Three aliphatic glucosinolates (GSs), *i.e.*, glucoiberin, gluconapin, glucobrassicanapin, and three indolic GSs, *i.e.*, glucobrassicin, 4-methoxyglucobrassicin, and neoglucobrassicin, were identified in both raw vegetables (Table 1). The aliphatic glucobrassicanapin was the most abundant GS in Chinese cabbage and pakchoi, accounting for about 48 and 63% of the total GSs, respectively, followed by glucoiberin for Chinese cabbage (31% of the total GSs) and gluconapin for pakchoi (16% of the total GSs). Meanwhile, the total content of indolic GSs contributes about 20 and 10% of the total GSs in Chinese cabbage and pakchoi, respectively. The total GS concentration in these two *Brassica rapa* vegetables is comparable with that reported in a previous studies but there are differences in the profile of major GSs [15–17]. Lewis and Fenwick [15] reported the main GSs in Chinese cabbage and pakchoi to be progoitrin, gluconapin, glucoalyssin, and glucobrassicanapin. In other studies, glucobrassicin and gluconasturtiin were also reported as major GSs in Chinese cabbage and pakchoi [16, 17]. Many factors, such as vegetable variety and environment, and method of analysis can influence the GS profile and concentration differences [16].

**Table 1:** Glucosinolates (μmol/g DW) in raw Chinese cabbage and pakchoi

	Chinese cabbage	Pakchoi
Glucoiberin	0.95 ± 0.08	0.17 ± 0.09
Gluconapin	0.07 ± 0.01	0.37 ± 0.05
Glucobrassicanapin	1.47 ± 0.16	1.46 ± 0.14
Glucobrassicin	0.24 ± 0.07	0.09 ± 0.02
4-Methoxy-glucobrassicin	0.29 ± 0.01	0.13 ± 0.02
Neoglucobrassicin	0.04 ± 0.01	0.07 ± 0.02
Total glucosinolates	3.07 ± 0.17	2.29 ± 0.16

Values are presented as mean ± standard deviation (*n* = 4)

### Effect of Stir-Frying

**Fig. 1:**
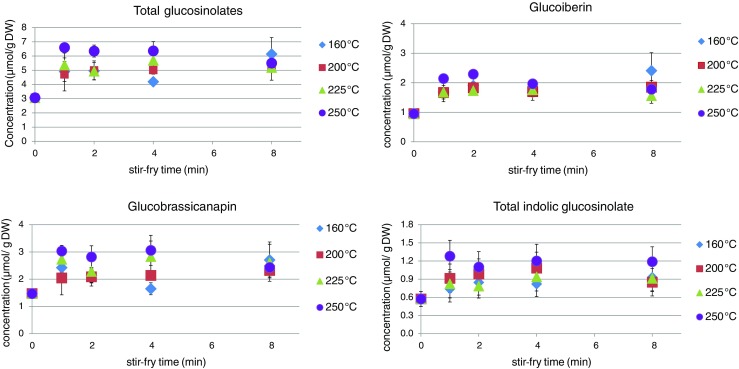
Total glucosinolates, glucoiberin, glucobrassicanapin, and total indolic glucosinolates (μmol/g DW) in Chinese cabbage during stir-frying. The legend shows the set stir-frying temperatures of the pan

**Fig. 2:**
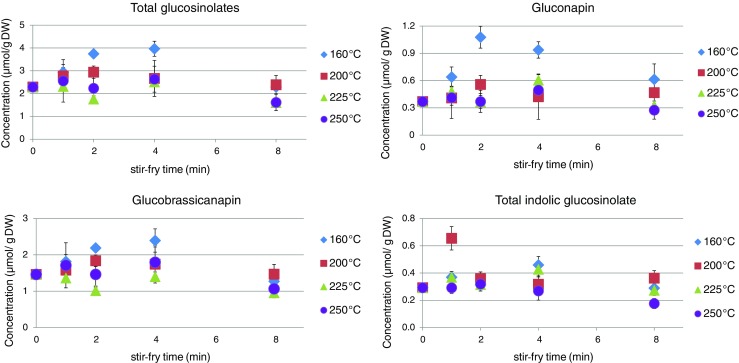
Total glucosinolates, gluconapin, glucobrassicanapin, and total indolic glucosinolates (μmol/g DW) in pakchoi during stir-frying. The legend shows the set stir-frying temperatures of the pan

The total GS content of Chinese cabbage after the first minute of stir-frying apparently increased by 30–100% compared to the content measured in the raw vegetable (Fig. 1, reported online, Table 2), and at 160, 200 and 250 °C the increasing was significant (Table 2). This trend was mainly caused by the aliphatic glucobrassicanapin and glucoiberin as the major GSs. Total indolic GSs only slightly increased after initial stir-frying (Fig. 1, reported online). Such increased concentration is also significantly different in the Chinese cabbage (Table 2). Different preparation methods have been reported to apparently increase the GS content after preparation, such as after microwave processing of red cabbage [14], boiling of cauliflower [18], and steaming of broccoli [19, 20]. It is likely that the heat treatment caused an increase in the extractability of GSs from the sample matrix during the analysis. A similar increase in total GS content was observed for pakchoi at temperatures 160 and 200 °C, however no significant difference was found in the total GS content (Fig. 2, Table 3). The different vegetable matrix may have affect the extractability of GSs, and it may be that the structure of the pakchoi is softer allowing a higher extraction yield. There were differences of GS thermal degradation between Chinese cabbage and pakchoi, *i.e.*, GSs in pakchoi were less stable and tend to decrease in accordance with the increase of cooking temperature. Significant differences were found at temperatures 160 and 200 °C between frying for 4 and 8 min in pakchoi samples that were not found for Chinese cabbage (Tables 2 and 3). Literature showed that the plant matrix in which the GSs are located, and its composition, could play an important role in the rate of GS degradation [7, 12, 13]. Hennig et al. [13], hypothesized that even different genotype and different growing conditions in the field, influence the thermal degradation rates of GSs. Moreover, the leafy physical structure of these two vegetables is different and such difference may affect the heat transfer during frying. Compared to Chinese cabbage, physically pakchoi has a more leafy structure and its leaves are thinner, so they are expected to heat up faster and to release more moisture during stir-frying. This moisture may contain GSs that are then exposed to the high temperature of the pan surface, resulting in a fast breakdown, especially at the highest temperature. Heat is transferred mostly by conduction from the hot surface of the pan or wok through a thin layer of hot oil. So, the surface temperature of vegetables rises rapidly and a proportion of water is vaporised [21]. Consequently, this rapid heating may have led to myrosinase inactivation. It was reported an 83% reduction in myrosinase activity in broccoli florets upon stir-frying for 4 min [9]. By microwave cooking for 4.8 min at 900 W, red cabbage reached 100 °C within the first minute of microwaving and upon this treatment myrosinase activity was not detected [22]. In the present study, since high frying temperatures were applied and the fact that thin strips of leaves were used, most of myrosinase enzyme from both vegetables was expected to be inactivated at the beginning of stir-frying, resulting in a negligible influence on the GS hydrolytic degradation. An effect of various times and temperatures during stir-frying on the GS content was not clearly seen in the present study. For both vegetables, the total, aliphatic, and indolic GS contents were relatively stable during stir-frying. During 8 min of stir-frying the GS contents in Chinese cabbage slightly increased, as compared to the changes of pakchoi. Moreover, higher set pan temperatures employed in the present study did not affect to the rate of GS changes. This can be explained by the fact that the temperature of a large part of the vegetable tissue would not exceed 100 °C due to the presence of water, resulting in the retention of GS. The studies performed on stir-frying of *Brassica* vegetables show considerable variation in the retention of GSs, mainly because different preparation and frying conditions were applied. A substantial retention of total aliphatic, indolic, and aromatic GSs in broccoli during stir-frying, even more than 95% as compared to the raw broccoli was observed [9]. They cooked 110 g broccoli florets in 5 mL oil for two minutes at oil temperature 80 °C, then added with 7 mL water followed by another stir frying for two minutes. Moreover, total GS content of stir-fried broccoli that was formerly blanched-and-frozen also showed no significant changes [9]. On the contrary, stir-frying of broccoli was reported to reduce the amount of the aliphatic and indolic GSs by about 55 and 67%, respectively [10]. More recently, stir-frying was reported to reduce 77% of total GSs in red cabbage [11]. These conflicting results might be due to specific stir-frying conditions applied and the different type of *Brassica* vegetable. Stir-frying experiments were performed at lower temperature (*i.e*., at 80 and 110–120 °C) and the cutting size of the vegetables was larger (florets 40 mm and strips 1 cm) [8, 9] than other experiments (*i.e*., piece of broccoli at 130–140 °C and 3 × 3 cm red cabbage at 130 °C) [10, 11]. In addition, different types of cooking oil were also found to influence to the loss of GS content [23]. Moreover, the loss of total GSs in broccoli by 84% was also reported when deep frying was applied apparently due to the intense thermal degradation [20]. The retention of GS can be explained by the lack of water addition during stir-frying. Leaching of GS has been reported to be one of the main factors that contributes to the reduction of GS contents during boiling and blanching of *Brassica* vegetables [24]. In a review on the mechanisms underlying GS changes during preparation, it was proposed that a low degree of leaching, thermal degradation, and enzymatic hydrolysis can be expected to occur during stir-frying of *Brassica* vegetables [5]. In the present study, at the applied time/temperature conditions, these mechanisms were indeed found to have a minimal effect on the GS, in fact the increased extractability of the GSs during stir-frying is the main responsible mechanism observed, especially in Chinese cabbage.

**Table 2:** Total glucosinolate concentration ± SD (μmol/g DW) during stir-frying in Chinese cabbage

Time (min)	160 °C	200 °C	225 °C	250 °C
0	3.07 ± 0.11 (a)	3.07 ± 0.11 (a)	3.07 ± 0.11 (a)	3.07 ± 0.11 (a)
1	4.91 ± 0.38 (bc)	4.71 ± 0.25 (b)	5.37 ± 1.00 (a)	6.60 ± 0.33 (b)
2	4.96 ± 0.08 (bc)	4.99 ± 0.50 (b)	4.93 ± 0.70 (a)	6.35 ± 0.10 (b)
4	4.19 ± 0.10 (ab)	5.01 ± 0.16 (b)	5.70 ± 1.17 (a)	6.37 ± 0.70 (b)
8	6.14 ± 0.88 (c)	5.13 ± 0.11 (b)	5.20 ± 0.78 (a)	5.50 ± 0.54 (b)

Different letters within a column indicate significant differences (*p* < 0.05)

**Table 3:** Total glucosinolate concentration ± SD (μmol/g DW) during stir-frying in pakchoi

Time (min)	160 °C	200 °C	225 °C	250 °C
0	2.29 ± 0.01 (a)	2.29 ± 0.01 (a)	2.29 ± 0.01 (ac)	2.29 ± 0.01 (a)
1	2.94 ± 0.41 (abd)	2.77 ± 0.44 (a)	2.31 ± 0.14 (ac)	2.56 ± 1.05 (a)
2	3.74 ± 0.06 (bc)	2.94 ± 0.28 (a)	1.76 ± 0.12 (bc)	2.23 ± 0.62 (a)
4	3.97 ± 0.10 (c)	2.67 ± 0.94 (a)	2.51 ± 0.20 (a)	2.63 ± 0.69 (a)
8	2.29 ± 0.19 (ad)	2.39 ± 0.48 (a)	1.60 ± 0.23 (b)	1.61 ± 0.44 (a)

Different letters within a column indicate significant differences (*p* < 0.05)

## Conclusion

In this study the retention of glucosinolates in Chinese cabbage (*Brassica rapa* ssp. *pekinensis*) and pakchoi (*Brassica rapa* ssp. *chinensis*) as affected by stir-frying at various times and temperatures is studied. The aliphatic glucosinolate glucobrassicanapin is the most abundant glucosinolate identified in raw Chinese cabbage. Upon the applied stir-frying processes, the concentration of all the glucosinolates did not decrease. The retention of glucosinolates is explained by (1) the lack leaching of these compounds into the cooking water, (2) the low thermal degradation rate of glucosinolate at those temperature/time conditions (3) the fact that the temperature in most of the plant tissue cannot exceed 100 °C (because of the water content) preventing the glucosinolate thermal degradation, and (4) the fast inactivation of myrosinase, preventing enzymatic glucosinolate hydrolysis during cooking. Therefore, a short stir-frying is a suitable cooking option to retain glucosinolate content in both vegetables, regardless the temperature applied. However, if for the Chinese cabbage 8 min of stir-frying did not affect the glucosinolate content, the glucosinolate content in the pakchoi was reduced for all the types of glucosinolate and temperatures applied. These results show that a short stir-frying, as cooking method, can be preferred over boiling for the retention of the water soluble glucosinolates.
